# Context-Aware Statistical Dead Reckoning for Localization in IoT Scenarios

**DOI:** 10.3390/s23135987

**Published:** 2023-06-28

**Authors:** David Munoz-Rodriguez, Rafaela Villalpando-Hernandez, Cesar Vargas-Rosales

**Affiliations:** Tecnologico de Monterrey, School of Engineering and Science, Monterrey 64849, Mexico; dmunoz@itesm.mx (D.M.-R.); cvargas@tec.mx (C.V.-R.)

**Keywords:** position location information, IoT, dead reckoning, stochastic dead reckoning, collaborative localization, context-aware localization

## Abstract

The current trends in 5G and 6G systems anticipate vast communication capabilities and the deployment of massive heterogeneous connectivity with more than a million internet of things (IoT) and other devices per square kilometer and up to ten million gadgets in 6G scenarios. In addition, the new generation of smart industries and the energy of things (EoT) context demand novel, reliable, energy-efficient network protocols involving massive sensor cooperation. Such scenarios impose new demands and opportunities to cope with the ever-growing cooperative dense ad hoc environments. Position location information (PLI) plays a crucial role as an enabler of several location-aware network protocols and applications. In this paper, we have proposed a novel context-aware statistical dead reckoning localization technique suitable for high dense cooperative sensor networks, where direct angle and distance estimations between peers are not required along the route, as in other dead reckoning-based localization approaches, but they are obtainable from the node’s context information. Validation of the proposed technique was assessed in several scenarios through simulations, achieving localization errors as low as 0.072 m for the worst case analyzed.

## 1. Introduction

Indoor and outdoor localization have re-gained relevance as an enabler of new applications and requirements introduced by IoT, EoT, Industry 4.0/5.0, and 5G/6G communications. For instance, the energy crisis has led to the EoT concept, which involves both industrial and domestic environments, as well as a widespread of smarter and more reliable scenarios in which energy consumption becomes a major issue and the deployment of energy-sensitive devices and systems will become even more relevant for static and dynamic applications.

Therefore, position location information (PLI) plays an important role in supporting the operation and management of mobile systems as it has become a crucial requirement for a growing and wide variety of location-based applications and services [1].

In scenarios where global navigation satellite systems (GNSS) are not available or their accuracy is compromised, most conventional position estimation systems consider a set of base stations as the reference sites, which are meant to have known locations, and they can be observed (or be seen) by a node of interest (NoI), whose coordinates need to be determined. In these scenarios, the node-to-landmarks distances can be estimated from a combination of parameters such as the received signal strength (RSS), time of arrival (ToA), time difference of arrival (TDoA), or angle of arrival (AoA) [2,3,4].

In ideal scenarios, simple range and angular observations may be sufficient to determine a 2D location. Those positioning setups are based on a direct observation of the radio source and are often referred to as single hop positioning systems. However, the presence of impairments precludes the operability of those systems, and several alternative trilateration schemes using multiple observation points are necessary.

In cases that lack a direct observation/connection and have limited power availability or unpredictable propagation scenarios, information delivery can be achieved via ad hoc node-to-node relays of transmitted information from/to, and many routing algorithms for node-to-node ad hoc paths selection can be found in the literature. Thus, PLI acquisition becomes cumbersome as conventional algorithms cannot be applied due to the lack of direct connectivity/observation.

This challenging problem becomes more relevant as the IoT and sensor networks become a pervasive experience, where densities as large as one million IP devices per square kilometer are anticipated for 5G and 6G systems.

In many cases, the device placement is imprecise, and it can be considered almost random; some probabilistic approaches have been suggested to support location acquisition processes, see [5,6,7].

The large number of IP devices, the mobility and connectivity changing scenarios, and the complexity of inhomogeneous propagation conditions cause uncertainty of the observable parameters. In order to have a reduced number of access points, low-cost alternative location techniques are demanded. Thus, context-aware information and collaborative location schemes have been suggested as promising techniques [8,9,10,11]. The use of cognitive approaches has also been suggested in order to acquire location information without a massive deployment of costly and bulky angle-range observation capabilities, and some relational schemes have also been proposed in the literature [12,13,14].

A dead reckoning approach usually depends on gyroscopic, accelerometer observations, the awareness of an initial reference location often called “a fix”, and on the knowledge of the consecutive traveling direction finding [10] of the consecutive steps, as well as the range or path length in each travel direction, in order to determine the progress of a traveler at a given time. Thus, conventional dead reckoning approaches demand highly equipped nodes with accurate angle/distance measurement capabilities [15,16].

In the IoT scenarios where tiny sensor nodes are deployed, localization techniques based on the angle of arrival observations and range measurements, as well as the implied network communication and processing cost, might not be a feasible approach [17].

In the EoT context, fault/attack sensor detection and localization techniques for photovoltaic (PV) systems have been addressed using statistical signal processing combined with machine learning techniques; however, there is a high processing cost involved in the use of artificial intelligence (AI) approaches [18]. In addition to the computational cost, these methods are not scalable for high dense networks.

In Table 1, we present a broad comparison between the position location techniques discussed above. This table compares several approaches in terms of the expected accuracy, network infrastructure (e.g., node equipment cost), processing cost, and environment suitability.

As noted before, the methods in [15,16] are based on the dead reckoning principle. The method reported in [15] provides an accurate location estimation of the nodes in ad hoc networks, and the reported estimation error is around [1.5 × 10^−2^ to 3.5 × 10^−2^]; however, they consider the time and angle of arrival estimation at each node in the route. Similarly, in [16], the authors propose a method based on previous measurements of the speed and acceleration of vehicles to calculate the current location using the dead reckoning approach; this implies that every vehicle in the network has measurement capabilities. The authors reported high location estimation errors.

In this paper, a novel dead reckoning approach based on the node context awareness and on the statistical characterization of the sensor node distribution is presented. The lack of the need for range/angle measurements equipment and processing makes the proposed technique adequate for the emerging localization paradigms. In this paper, we assume that source-to-destination node paths in a high-density scenario can be established. Nevertheless, the routing algorithm is unsubstantial for the purposes of this paper. Although the current technological trends include enhanced beam-forming capabilities [19], these are not required. However, at the initial point of an ad hoc path, we also show that the direction and range of successive steps can be obtained from the node distribution. The feasibility of the proposed algorithm is assessed in several scenarios in Section 3 based on Figures 4–7, where the accuracy of the method is evaluated under different network parameters, such as the node coverage range and node density.

Please note that the proposed method is partially based on examinations performed as part of thesis research work, as reported in [20].

## 2. Context-Aware Statistical Dead Reckoning Approach

In the case of Ad Hoc and Wireless Sensor Networks (AH-WSNs), direct connection between the NoI (node of interest) and base stations or anchor point (AP) may be unavailable. This lack of direct connectivity precludes the use of direct trilateration schemes. However, connectivity is established via the concatenation of consecutive links or hops. The localization of a NoI is obtained through the addition of successive vectors corresponding to the link hops. This demands that the hop lengths and physical directions be known [15,16].

APs are assumed to have fixed and known coordinates and they provide access to the rest of the telecommunication network. Several location and angular finding schemes for ad hoc scenarios have been proposed in the literature [21,22,23,24,25,26]. However, the reduced size, power availability constraints, and limited processing capabilities of most nodes make the deployment of direction-finding schemes unviable for the non-anchor nodes. In contrast, APs are assumed to have larger power budgets, higher reception sensitivity, and enhanced processing capabilities in order to be suited for direction finding deployment. In this paper, it is shown that context-aware information allows the application of dead reckoning techniques without a massive deployment of angle finding and direct range measurements, while in traditional schemes, such as those reported in [15,16], several distance/angle measurements are required.

The fast growth of sensor and ad hoc networks has already been noted, and this approach takes advantage of the large number of nodes deployed. In this paper, the proposed methodology is applicable for 2D scenarios; however, it can be generalized for 3D scenarios.

Let us consider a sensor network in which each node is aware of their one hop reachable nodes. Let ***n****_i_*, *i* = 1, 2, …, be a node with coordinates (*x_i_*, *y_i_*) and reachability radius *R_i_*. We define an *R_i_*-neighborhood or an *R_i_*-ball, **B**(*R_i_*) as the set of reachable nodes ***n****_ζ_* with coordinates (*x_ζ_*, *y_ζ_*) within its coverage radius *R_i_*; that is, **Β**(*R_i_*) = {***n****_ζ_*│(*x_ζ_* − *x_i_*)^2^ + (*y_ζ_* − *y_i_*)^2^ ≤ *R_i_*^2^}. Note that the coverage range *R_i_* is set by the sensitivity of the receiver/transmitter systems and transmitted power.

**Figure 1:**
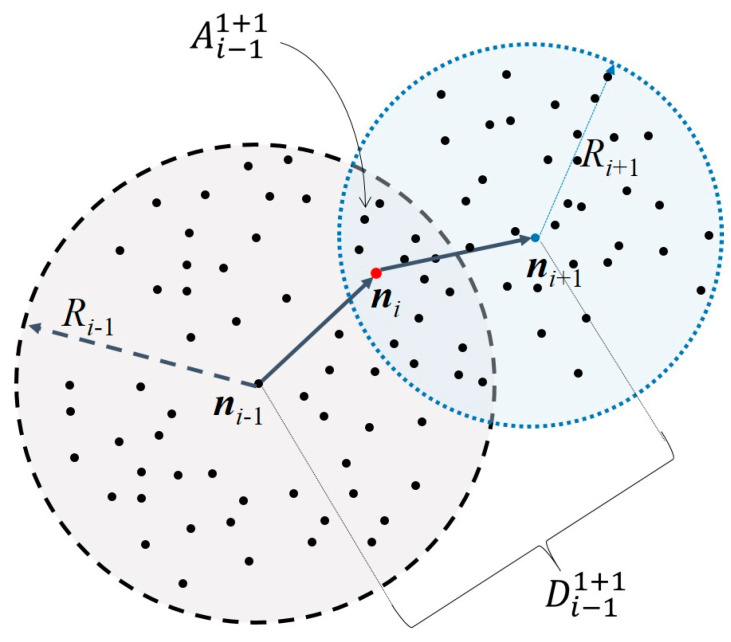
Intersection of **B**(*R_i_*_−1_) with **B**(*R_i_*_+1_).

For an ordered sequence of nodes ***n****_i_*_−1_, ***n****_i_*, ***n****_i_*_+1_ in an ad hoc path, node ***n****_i_* is meant to be reachable from both ***n****_i_*_−1_ and ***n****_i_*_+1_, this is ***n****_i_*∈ **B**(*R_i_*_−1_) ∩ **B**(*R_i_*_+1_), as illustrated in Figure 1. For known reachability radiuses *R_i_*_−1_ and *R_i_*_+1_, the area $A_{i-1}^{i+1}$ of the intersection of **B**(*R_i_*_−1_) ∩ **B**(*R_i_*_+1_) is a function of the separation distance—$D_{i-1}^{i+1}=\sqrt{{\left(x_{i-1}-x_{i+1}\right)}^{2}+{\left(y_{i-1}-y_{i+1}\right)}^{2}}=D_{i+1}^{i-1}$—of nodes ***n****_i_*_−1_ and ***n****_i_*_+1_, and, as a matter of fact, it can be shown (see [27]) that these parameters relate, according to:

(1) $$\begin{array}{c} A_{i-1}^{i+1}=f\left(D_{i-1}^{i+1},R_{i-1},R_{i+1}\right)\\ =R_{i-1}^{2}\left\{\arccos\left(\frac{\alpha }{2D_{i-1}^{i+1}R_{i-1}}\right)-\left(\frac{\alpha }{4{D_{i-1}^{i+1}}^{2}R_{i-1}^{2}}\right)\sqrt{4{D_{i-1}^{i+1}}^{2}R_{i-1}^{2}-{\left(\alpha \right)}^{2}}\right\}\\ +R_{i+1}^{2}\left\{\arccos\left(\frac{\gamma }{2D_{i-1}^{i+1}R_{i+1}}\right)-\left(\frac{\gamma }{4{D_{i-1}^{i+1}}^{2}R_{i+1}^{2}}\right)\sqrt{4{D_{i-1}^{i+1}}^{2}R_{i+1}^{2}-{\left(\gamma \right)}^{2}}\right\}, \end{array}$$

where $\alpha ={D_{i-1}^{i+1}}^{2}+R_{i-1}^{2}-R_{i+1}^{2}$ and $\gamma ={D_{i-1}^{i+1}}^{2}+R_{i+1}^{2}-R_{i-1}^{2}$. Note that $A_{i-1}^{i+1}$ in (1) is a monotonic function of the separation distance $D_{i-1}^{i+1}$. Therefore, for given reachability radiuses *R_i_*_−1_, *R_i_*_+1_, $D_{i-1}^{i+1}$ can be inferred from $A_{i-1}^{i+1}$. As expressions involve transcendental functions, inversion becomes cumbersome, but it can be obtained numerically. In addition, note that for equal reachability radiuses, i.e., $R_{i-1}=R_{i+1}$, Equation (1) is reduced to:

(2) $$A_{i-1}^{i+1}=2{R_{i-1}}^{2}\arccos\left(\frac{D_{i-1}^{i+1}}{2R_{i-1}}\right)-\frac{1}{2}D_{i-1}^{i+1}\sqrt{4{R_{i-1}}^{2}-{D_{i-1}^{i+1}}^{2}},$$

**Figure 2:**
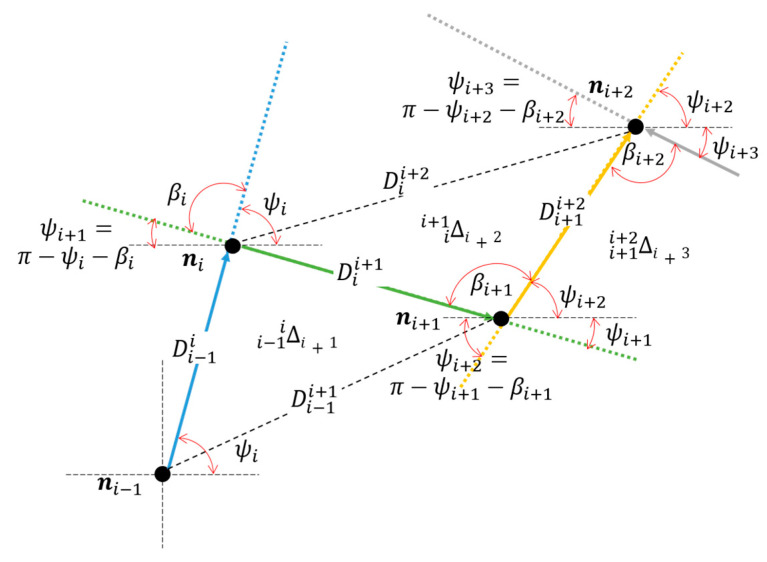
Construction of the Dead reckoning path from node ***n****_i_*_−1_ to node ***n****_i_*_+2_.

For instance, in a two-dimensional scenario, for the path segment ***n****_i_*_−1_,***n**_i_*, ***n****_i+_*_1_, $D_{i-1}^{i}$, and $D_{i}^{i+1}$ denote, respectively, the separation of consecutive nodes—***n****_i_*_−1_,***n**_i_*, and ***n****_i_*, ***n****_i+_*_1_—that can be estimated from the field strength or delay measurements. On the other hand, nodes ***n****_i_*_−1_, ***n****_i+_*_1_ are not adjacent, but their separation $D_{i-1}^{i+1}$ can be inferred from $A_{i-1}^{i+1}$ and, consequently, a triangle Δ($D_{i-1}^{i}$, $D_{i-1}^{i+1},D_{i}^{i+1}$) = Δi+1i−1i can be defined by its side lengths, and such a triangle contains a segment ***n****_i−_*_1_, ***n****_i_*, ***n****_i+_*_1_ of a dead reckoning path that links an anchor to a NoI. A similar process can be conducted for nodes ***n****_i_*, ***n****_i_*_+1_, and ***n****_i_*_+2_, and a triangle Δi+2ii+1 can be constructed. In addition, it can be seen that side $D_{i}^{i+1}$ is common to both triangles (see Figure 2).

Dead reckoning is the process of inferring a location using solely consecutive distances $D_{i-1}^{i+1}$ and step direction estimates from a reference known site (*x*_0_, *y*_0_) or fix. Assuming the fix is at an anchor location (*x*_0_, *y*_0_) and that nodes ***n****_i_* in a path occupy coordinates (*x_i,_*, *y_i_*), *i* = 1, 2, 3, …, the location of node ***n****_n_* placed at (*x_n_*, *y_n_*) can be figured out as the vector addition $\rho =\sum_{i=1}^{n}\rho_{i}$, where $\rho_{i}=\left(\rho_{i},\psi_{i}\right)$ is defined by $\rho_{i}=\sqrt{{\left(x_{i}-x_{i-1}\right)}^{2}+{\left(y_{i}-y_{i-1}\right)}^{2}}$ and $\psi_{i}=\arctan\frac{y_{i}-y_{i-1}}{x_{i}-x_{i-1}}$ or $\psi_{i}={tan}^{-1}\frac{y_{i}-y_{i-1}}{x_{i}-x_{i-1}}$, i.e., $\rho_{i}$ is the range and $\psi_{i}$ is the azimuthal angle. At this stage, coordinates (*x_i_*, *y_i_*) are unknown, with the exception of (*x*_0_, *y*_0_).

We pointed out that the range estimates $\rho_{i}$ are obtainable through the field strength or delay measurements. However, angular observations tend to be bulkier and resource consuming. Nevertheless, anchor points are more able in terms of available energy, antenna capabilities, and computing power. Therefore, the initial angle $\psi_{0}$ is considered to be known and observable at the anchor point. The subsequent angular directions $\psi_{i}$, *i* = 1,2, …, are inferred from the node context as it is explained in the following paragraphs.

Recall that an ad hoc path is formed by consecutive hops connecting nodes ***n***_0_, ***n***_1_,…***n****_i_*_−1_, ***n****_i_*, ***n****_i_*_+1_, ***n****_i_*_+2_, …, ***n****_N_*. It can be observed that a path is contained within the sides of the concatenation of adjacent triangles Δ201, Δ312, …, Δi+1i−1i, Δi+2ii+1, …, ΔNN−2N−1 and the triangle concatenation allows the construction of a dead reckoning path.

Consider a homogeneous scenario with randomly deployed nodes to obtain $A_{i-1}^{i+1},$ which is the area estimation of the intersection of **B**(*R_i−_*_1_)∩**B**(*R_i_*_+1_). The number *N* of nodes in an area *A* is distributed according to Poisson as $\frac{{\left(\lambda A\right)}^{N}}{N!}e^{-\lambda A}$, where λ stands for the node density (nodes per area) parameter, and for a given number *N* of nodes*,* the maximum probability occurs for λA=N [28]. Thus, for a known node density λ, the estimation of the area $A_{i-1}^{i+1}$ can be obtained from the number *N* of nodes in the intersection **B**(*R_i_*_−1_) ∩ **B**(*R_i_*_+1_).

In practice, the node density λ is not known, but it can be approximated from the average number of nodes $N_{i-1}$ and $N_{i+1}$ in balls **B**(*R_i−_*_1_) and **B**(*R_i_*_+1_), respectively. For instance, the estimated node density $\lambda^{*}$ is given by: $\lambda^{*}\approx \left[\frac{N_{i-1}}{R_{i-1}^{2}}+\frac{N_{i+1}}{R_{i+1}^{2}}\right]/4\pi .$

It has been stated that the location of all the nodes in the ad hoc path become known after the initial hop ***n***_0_ − ***n***_1_ is known, where ***n***_0_ is an anchor node with enhanced angle observation capabilities. Subsequent angular estimations can be obtained via the node context. Note that angle $\beta_{i}$ subtended by adjacent hop lines of length $D_{i-1}^{i}$ and $D_{i}^{i+1}$ (see Figure 2) is given by:

(3) $$\beta_{i}=\mathrm{acos}\left[\frac{{\left(D_{i-1}^{i}\right)}^{2}+{\left(D_{i}^{i+1}\right)}^{2}-{\left(D_{i-1}^{i+1}\right)}^{2}}{2D_{i-1}^{i}D_{i}^{i+1}}\right].$$

This scheme produces a vector sequence, $\rho_{i}=\left(\rho_{i},\psi_{i}\right),$ which allows us to construct a dead reckoning path as the vector addition $\rho =n_{0}+\sum_{i=1}^{n}\rho_{i},$ where node $n_{0}$ is meant to be an anchor node that has direction finding capabilities and known coordinates. Thus, $\rho_{1}=\left(\rho_{1},\psi_{1}\right)$ is known and the subsequent vectors $\rho_{i}=\left(\rho_{i},\psi_{i}\right),i=2,\ldots$, are defined by the measurements of $\rho_{i}=D_{i-1}^{i}$ and calculations $\psi_{i}=\pi -\psi_{i-1}-\beta_{i-1}$.

**Figure 3:**
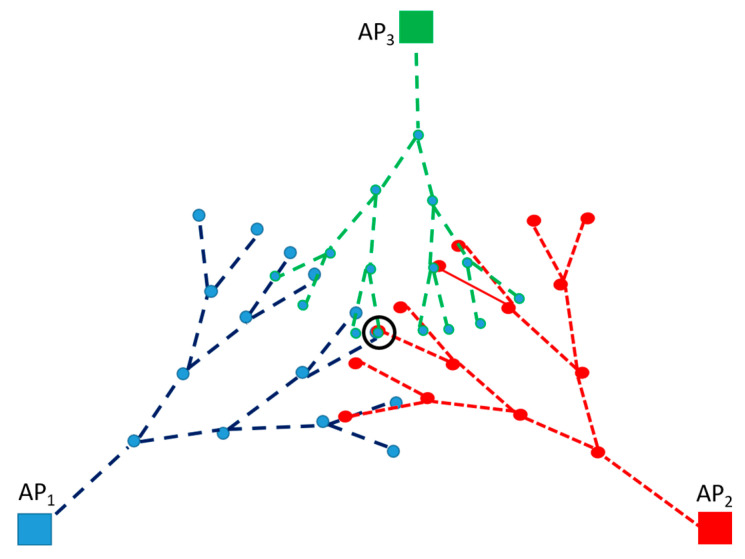
Intersection of spanning trees using dead reckoning paths.

Note that for the triangle, $\Delta =\left(D_{0}^{1},D_{0}^{2},D_{1}^{2}\right)$, the placement of the edge $D_{0}^{1}$ is unique. However, there are two specular triangles with that common edge. This leads to two options for the location of ***n***_2_. This ambiguity grows as a spanning tree as the path progresses towards the node of interest NoI. Nevertheless, the uncertainties are removed by taking the intersection of the spanning trees with roots at different anchor points (see Figure 3).

Equation (1) states that $A_{i-1}^{i+1}=f\left(D_{i-1}^{i+1},R_{i-1},R_{i+1}\right).$ As it is a monotonic function of $D_{i-1}^{i+1}$, for given reachability radiuses *R_i_*_−1_ and *R_i_*_+1_, $D_{i-1}^{i+1}$ can be inferred from $A_{i-1}^{i+1}$ through a minimization search function, given by:

(4) $$\tilde{D_{i-1}^{i+1}}=\underset{D_{i-1}^{i+1}}{\min}\left\{{\left[f\left(D_{i-1}^{i+1},R_{i-1},R_{i+1}\right)-A_{i-1}^{i+1}\right]}^{2}\right\},$$

Error drifts tend to accumulate along the path. Nevertheless, in this paper, it is shown that the multilateration of the reckoning paths allows for a location uncertainty reduction.

As stated previously, the proposed localization method can be extended for 3D scenarios. However, several considerations must be made. First, we will have the intersection of two spheres instead of circles (Figure 1). Second, distance $D_{i-1}^{i+1}$ must be redefined to include the “*z*” coordinate. Third, this would force it to have an intersection volume, and Equations (1)–(3) would be reformulated for a 3D geometry. Additionally, the node density estimation equation must be reformulated under the basis of the intersection volumes. Finally, the minimization search function in Equation (4) must also be reformulated to perform the search on the intersection volume.

## 3. Simulations and Results

In order to assess the feasibility of the algorithm, different homogenous scenarios are created where the access points are assumed to be evenly spaced in a circle of radius *R_A_* that defines the coverage area *A*. Note that *R_A_* = *R_i_*_−1_ = *R_i_*_+1_ for all *i*. The obtained location is compared to the actual NoI coordinates (*x*, *y*) by calculating the error according to $\varepsilon^{2}={\left(x-x^{*}\right)}^{2}+{\left(y-y^{*}\right)}^{2}$. That is, we summarize the simulation process as follows:

1. For a given scenario, the number of nodes *N_i_*_−1_ and *N_i_*_+1_ within each of the coverage balls **B**(*R_i_*_−1_) and **B**(*R_i_*_+1_) corresponding to nodes *n_i_*_−1_ and *n_i_*_+1_ are known. Then, λ^*^ can be obtained.
2. Once λ^*^ is estimated and the number of nodes *N* at the intersection is known, it is possible to estimate the intersection area $A_{i-1}^{i+1}$.
3. From $A_{i-1}^{i+1}$, *R_i_*_−1_, and *R_i_*_+1_, we can calculate the estimated separation distance $\tilde{D_{i-1}^{i+1}}$ using the minimization problem solution from (4).
4. Error will be assessed through a comparison of the estimated distance $\tilde{D_{i-1}^{i+1}}$ versus the actual separation distance $D_{i-1}^{i+1}$.
5. We repeat this process depending on the number of hops required to reach the node of interest, NoI.

**Figure 4:**
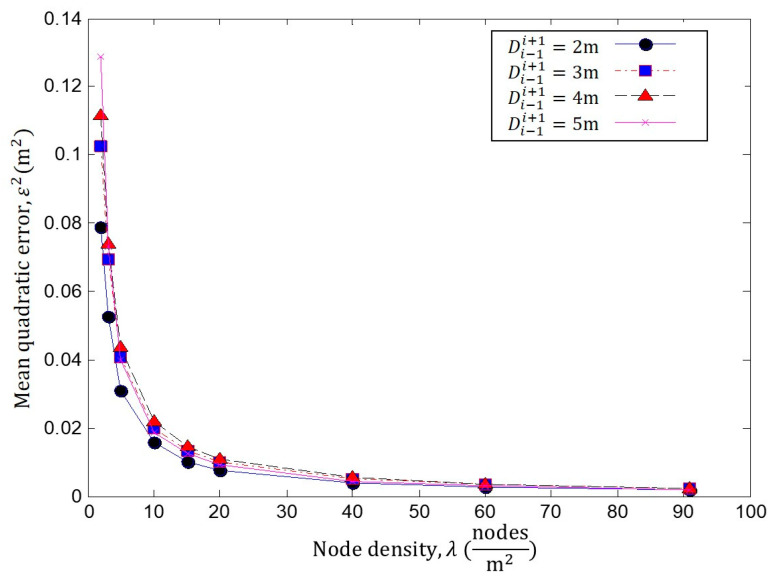
Performance analysis *λ* against $\varepsilon^{2}$ for different $D_{i-1}^{i+1}$, with *R_A_* = 3 m.

In the following simulations, we consider a scenario, such as in Figure 1, where the NoI is n* = *n_i_*. The accuracy ($\varepsilon^{2}$) of the proposed localization technique is evaluated for different node densities *λ*, coverage radiuses *R*_A_, as well as for different separation distances $D_{i-1}^{i+1}$. Figure 4 shows the performance of the method under errors due to the difference in the current *λ* and the estimated λ^*^ node densities and to the difference between the true $D_{i-1}^{i+1}$ and estimated $\tilde{D_{i-1}^{i+1}}$ separation distances for *R_A_* = 3 m.

**Figure 5:**
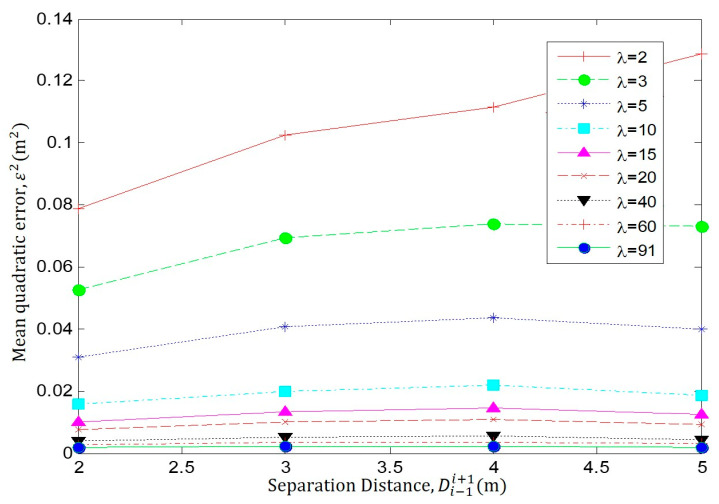
Performance analysis $D_{i-1}^{i+1}$ against $\varepsilon^{2}$ for different *λ*, with *R_A_* = 3 m.

**Figure 6:**
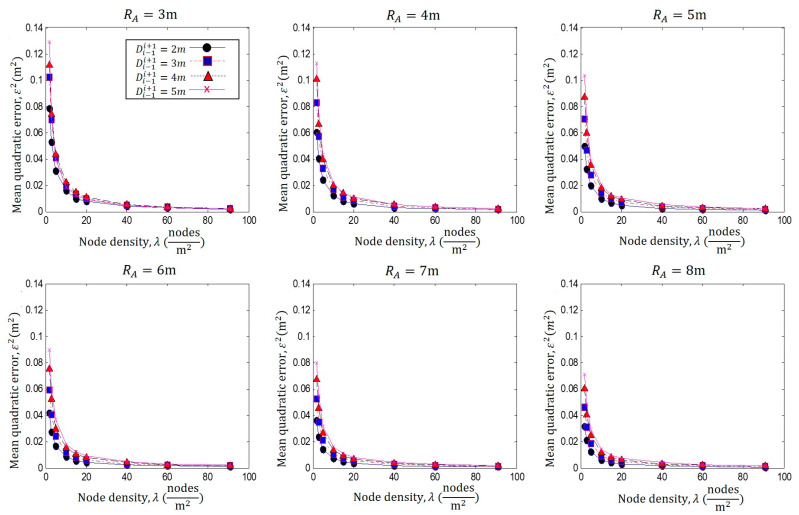
Performance analysis *λ* against $\varepsilon^{2}$ for different $D_{i-1}^{i+1}$, with *R_A_* = 3 m to 8 m.

In Figure 5, we compare the localization error *ε*^2^ for different node densities *λ* = (2, 3, 5, 10, 15, 20, 40, 60 and 91) when *R_A_* = 3. Each curve represents the localization error *ε*^2^ at different separation distances $D_{i-1}^{i+1}$, ranging between 2 and 5 m. It is possible to observe how the error increases as the separation distance grows when the density is low. In addition, the error decreases due to the node density *λ* increments, as these increments provide improvements in the intersection area. In Figure 6, we present the same results under a different perspective.

In Figure 5, it is clear that *ε*^2^ decreases as increments of *λ* provide improvements in the estimation of the intersection area. In addition, *ε*^2^ increases as $D_{i-1}^{i+1}$ grows. These are the expected results, as the localization method depends on the estimated λ^*^ and $\tilde{D_{i-1}^{i+1}}$. Please note that despite the fact that the simulations are commanded to prove the true parameters (*λ* and $D_{i-1}^{i+1}$), the localization method estimates the NoI position using estimated parameters (λ^*^ and $\tilde{D_{i-1}^{i+1}}$), as stated above.

In Figure 6, the same analysis is realized for several coverage ranges of *R_A_* = 3 to 8 m, plotting the mean quadratic error $\varepsilon^{2}$ vs. *λ*. In this figure, we can observe that each of the graphs presents the same analysis as that in Figure 4, i.e., the localization error *ε*^2^ increases as the separation distance $D_{i-1}^{i+1}$ is increased.

However, it is also noticeable that as the size of the coverage radio *R_A_* increases, the localization error *ε*^2^ decreases. This is because the greater the intersection area, the greater the number of nodes at the intersection and the better the approximation; therefore, *ε*^2^ is decreased.

**Figure 7:**
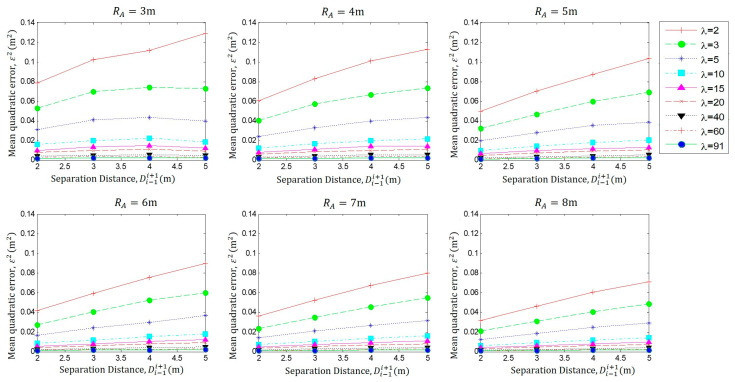
Performance analysis $D_{i-1}^{i+1}$ against $\varepsilon^{2}$ for different *λ*, with *R_A_* = 3 m to 8 m.

In Figure 7, we present the same data representation as that in Figure 5 to provide a better understanding. From this data observation, it should be noted that the localization error *ε*^2^ is inversely proportional to the coverage range *R_A_*.

As we established earlier, dead reckoning does not require a large node density to obtain accurate results. However, if we compare the results, we can see that the decrease in the quality of the estimation is minimal between a low-density user scenario and a large-density one. It is also important to note that the last three values of user density appear to behave in the same way regardless of the size of the coverage range.

Please note that even though the initial consideration was for a scenario such as that illustrated in Figure 1, this methodology can be repeated for several hops to apply in scenarios such as that in Figure 3.

## 4. Conclusions

In this paper, it is shown that context information and the number of observable neighboring nodes allow the position location problem in dense ad hoc IoT scenarios to be solved without a high increase in the node’s equipment. A stochastic dead reckoning methodology is presented and the proof of concept is demonstrated using simulations. The proposed algorithm estimates the local node density and constructs the feasible dead reckoning paths that converge to determine the actual location of a NoI. Enhanced adequate processing and direction-finding capabilities are only required at anchor nodes, and these are not required for the remaining nodes that can estimate the lengths of the adjacent edges in the path from the field strength or delay measurements. This allows concatenations of triangles to be built that contain ad hoc paths that lead to the location inference of the NoIs. Performance results are reported for several scenarios, and it is shown that the proposed context-aware statistical dead reckoning method offers a good performance under several network conditions, such as coverage range, separation distance, and node density (i.e., *ε*^2^ < 0.072 for worst case using a coverage range *R_i_* = 8 m). The main achievements of the proposed method can be identified as follows:

- The method does not impose processing burn out on nodes.
- The method only requires angle measurement capabilities on anchor nodes.
- The method is based on easily obtainable network information (i.e., number of neighbors).
- The method provides an accuracy of *ε*^2^ = 0.13 m and *ε*^2^ = 0.072 m for *R_i_* = 3 m and *R_i_* = 8 m, respectively, for the worst cases analyzed.

Future analysis can be conducted for diverse network conditions and for randomly selected routes considering nodes with heterogeneous capabilities, including performance with non-uniform node densities.

## Figures and Tables

**Table 1:** Location Techniques Comparison.

Principle	Network Infrastructure	Processing Cost	Scenario	Accuracy
Context-aware and collaboration location techniques [8,9,10,11].	Low equipped. Cognitive-based approaches, distance-based.	High	Low dense networks	Low
Relational-based approaches [12,13,14].	Low equipped ANs and nodes. Hop-count-based.	Low	High dense networks	Low
Dead Reckoning-based approaches [15,16].	High equipped ANs and nodes. Range/Angle-based.	High	High dense networks	High

## Data Availability

Not applicable.
